# Predictive utility of commercial grade technologies for assessing musculoskeletal injury risk in US Marine Corps Officer candidates

**DOI:** 10.3389/fphys.2023.1088813

**Published:** 2023-01-17

**Authors:** Matthew B. Bird, Kristen J. Koltun, Qi Mi, Mita Lovalekar, Brian J. Martin, Tim L. A. Doyle, Bradley C. Nindl

**Affiliations:** ^1^ Department of Sports Medicine and Nutrition, Neuromuscular Research Laboratory/Warrior Human Performance Research Center, University of Pittsburgh, Pittsburgh, PA, United States; ^2^ Department of Health Sciences, Biomechanics, Physical Performance and Exercise Research Group, Macquarie University, Sydney, NSW, Australia

**Keywords:** machine learning, supervised learning, military, random forest, recursive partitioning

## Abstract

Recently, commercial grade technologies have provided black box algorithms potentially relating to musculoskeletal injury (MSKI) risk and functional movement deficits, in which may add value to a high-performance model. Thus, the purpose of this manuscript was to evaluate composite and component scores from commercial grade technologies associations to MSKI risk in Marine Officer Candidates. 689 candidates (Male candidates = 566, Female candidates = 123) performed counter movement jumps on SPARTA™ force plates and functional movements (squats, jumps, lunges) in DARI™ markerless motion capture at the start of Officer Candidates School (OCS). De-identified MSKI data was acquired from internal OCS reports for those who presented to the Physical Therapy department for MSKI treatment during the 10 weeks of training. Logistic regression analyses were conducted to validate the utility of the composite scores and supervised machine learning algorithms were deployed to create a population specific model on the normalized component variables in SPARTA™ and DARI™. Common MSKI risk factors (cMSKI) such as older age, slower run times, and females were associated with greater MSKI risk. Composite scores were significantly associated with MSKI, although the area under the curve (AUC) demonstrated poor discrimination (AUC = .55–.57). When supervised machine learning algorithms were trained on the normalized component variables and cMSKI variables, the overall training models performed well, but when the training models were tested on the testing data the models classified MSKI “by chance” (testing AUC avg = .55–.57) across all models. Composite scores and component population specific models were poor predictors of MSKI in candidates. While cMSKI, SPARTA™, and DARI™ models performed similarly, this study does not dismiss the use of commercial technologies but questions the utility of a singular screening task to predict MSKI over 10 weeks. Further investigations should evaluate occupation specific screening, serial measurements, and/or load exposure for creating MSKI risk models.

## Introduction

A high-performance model for sport is a program in which the training environment is modeled for the success of the athlete by utilizing support staff (e.g., athletic trainers, strength coaches), facilities, and athlete monitoring (e.g., force plates, online questionnaires) (Turner et al., 2019). Commercially available devices have also been integrated into these high-performance environments in Olympic, professional, and National Collegiate Athletic Association (NCAA) athletics to assess overall performance and screen for musculoskeletal injury (MSKI) (Smith and Smolianov, 2016; Turner et al., 2019). For example, force plates and GPS tracking have been used to monitor athletes’ fatigue and readiness that may contribute to their success in sport (Gathercole et al., 2015; Hulin et al., 2016). Calculating training load (e.g., distance covered through GPS), and incorporation into models such as the acute:chronic workload ratio, have been developed to provide a single answer solution to evaluate fatigue based on continuous monitoring technology (Hulin et al., 2016). Additionally, serial measurements with devices such as force plates, have been implemented to assess for neuromuscular readiness for performance (Cormack et al., 2008). Typically, these measures are assessed to provide trends, which are calculated and monitored during regular season play to indicate fatigue associating to MSKI risk. With multiple domains of technology contributing to a plethora of data source outcomes, support staff, such as sport scientists, athletic trainers, and/or strength coaches, are necessary to analyze and interpret results for coaches. For the few programs that have the budget, resources, and support staff, there are many more that do not have access to these accommodations. Thus, commercial grade companies have invested in technology that can analyze and package the information (i.e., MKSI risk) in seconds after testing. In theory, a single technology that could accurately display MSKI risk and performance readiness would mitigate the need for back-end processing and create efficiencies for support staff, thereby increasing its utility among populations and settings. As such, professional and NCAA sports teams, and the military have leveraged these commercially available technologies for their field deployability and ease of implementation (Parisien et al., 2021).

Due to the large number of armed services members located across the United States and globally, this high-performance approach is challenging and complex to implement efficiently in military populations. For example, in the United States, the Army has 31 bases, with 378,900 active component soldiers (APHC, 2020). Despite this challenge, it is necessary due to the sheer amount of MSKIs that occur in Service members, in which over 50% of Army soldiers sustain a MSKI resulting in 2 million medical encounters (APHC, 2020). To combat the high rate of MSKIs, the Army has implemented the Holistic Health and Fitness (H2F), that is, intended to broaden the physical fitness attributes of soldiers with the assistance of performance coaches and facilities/equipment (Molloy et al., 2020). Additionally, with the heightened push for screening to detect MSKIs, Congress has mandated that evidence be provided to support the use of force plates combined with machine learning to help mitigate MSKIs (Thornberry, 2020). As such, a technology, that is, field deployable and expedient, and which can accurately demonstrate MSKI risk, would have great utility across all Services to further reduce the burden of MSKI.

Commercial grade force plates (FP) are an emerging technology that may provide useful data for MSKI risk associations. Recently, data extracted from the performance of counter movement jumps (CMJs) performed on SPARTA™ force plates have been used to predict ACL injuries (Pontillo et al., 2021) and elbow injuries (Mayberry et al., 2020), and to mitigate athlete healthcare costs (Parisien et al., 2021). Parisien et al. (2021), reported the implementation of the CMJs *via* SPARTA™ FP and saw no significant injury difference in those that utilized SPARTA™ force plates than those that did not, while the injury-related healthcare costs were significantly higher in the non-user group. In addition, Pontillo et al. (2021), reported SPARTA™ FP variables (Explode and Drive) were predictive of ACL injury over a 10-week exposure. It is important to note, these studies used measures derived from the CMJ *via* SPARTA™ (i.e., Load, Explode, and Drive), which are derivatives of common force place variables and did not report the SPARTA™ MSKI prediction algorithms (i.e., MSKI Health and Risk Group). Alternatively, Hando et al. (2022) evaluated the MSKI Health composite score and other SPARTA™ measures in special warfare trainees and found the MSKI Health score was not predictive of MSKI [OR (95% CI) = .986 (.956–1.016)].

Another emerging technology, that is, gaining popularity due to its automation of objective data inputs from a movement screen is markerless motion capture (mMoCap). mMoCap addresses previous limitations of marker-based motion capture (MoCap), the gold standard to evaluate kinetics and kinematics, that is, largely constrained to state of the art, biomechanical facilities. mMoCap has been investigated in healthcare (Martinez et al., 2018) and athletics (Sonnenfeld et al., 2021) and proven to be reliable (Mosier et al., 2018; Drazan et al., 2021) and valid (Perrott et al., 2017). DARI™ mMoCap, a commercial grade mMoCap system, developed a “Joint Quality” composite score in which each joint is normalized and scored based on DARI’s™ internal database, to indicate if a joint is outside normative ranges during common movements such as body weight squats, CMJs, drop jumps, and single leg CMJs. Recently, Hando et al. (2021) calculated odds ratios utilizing the overall vulnerability score for MSKI (OR = 1.01) and lower extremity MSKI (OR = 1.02) in military trainees (*n* = 1,540) and determined the overall vulnerability score was not a clinically useful measure to predict MSKI.

As the military pushes a for a reduced MSKI burden with quick actionable decision aids, these tools could add value in accomplishing this mission. Hando et al. (2021); Hando et al. (2022) is the only group to have examined the utility of the DARI™ (i.e., Joint Quality) and the SPARTA™ (i.e., MSKI Health) composite scores for associations with MSKI. While they have reported poor utility of the composite scores to associate MSKI risk in Airforce trainees, it is as yet unclear if SPARTA™ and DARI™ models will carry over to a different military population. Similar to Airforce trainees, Marine Officers candidates undergo training pipelines (Officer Candidates School) with a high MSKI incidence rate (Piantanida et al., 2000). While Hando et al. only reported male Airforce trainees, Officer Candidates School consists of male and female candidates, in which the SPARTA™ and DARI™ MSKI models may have better predictive utility when females are included. Thus, the present study sought to evaluate the SPARTA™ and DARI™ composite scores in male and female Marine Officer Candidates in association with lower extremity and torso MSKI’s during 10-week of Marine Corps Officer Candidates training. Additionally, we assessed the SPARTA™ and DARI™ normalized component variables and common MSKI risk factor variables (cMSKI) to see if a population specific model would increase the predictive utility of SPARTA™ and DARI™.

## Materials and methods

Researchers briefed and consented Marine Officer candidates for the study. Ethical approval was provided by the University of Pittsburgh Institutional Review Board (STUDY19030386) and the research was endorsed by the Office of Naval Research and Officer Candidates School (OCS). A total of 689 candidates (Female candidates = 123, Male candidates = 566) comprising four intake classes signed informed consents and participated in the DARI™ and SPARTA™ testing.

### Officer Candidates School

OCS is a 10-week military training course designed for individuals seeking to become commissioned officers in the United States Marine Corps. OCS consists of controlled daily physical and military training, along with graded events that test for aerobic capacity (i.e., 3-mile run), obstacle navigation and loaded ruck marches. All candidates are required to do the same training regardless of job (e.g., attorney, infantry officer, intelligence), and sex, and there are high incidences of lower extremity and torso MSKIs that occur [Male candidates = 23% and Female candidates = 36% (Bird et al., 2022)].

### Movement assessment

Prior to the start of physical training, height and mass were recorded by a stadiometer and digital scale (Healthometer Professional 500KL, McCook, IL). Self-reported questionnaires regarding prior MSKI (retrospective 1 year), were administered *via* the Research Electronic Data Capture (RedCap) on an electronic tablet. Candidates were required to perform a warm-up and familiarization phase consisting of the SPARTA™ FPs and DARI™ mMoCap movements prior to testing.

SPARTA Science™ FP (SPARTA Science™, California), sampling at 1,000 Hz, were used for data collection. Candidates performed three maximal-effort CMJs, with ∼15 s rest (pre-determined in SPARTA™ software) between each jump. The candidates were cued to start with hands above head, stand still (1 s of quiet phase to register system mass), and performed the jump with a counter-movement and arm swing to a self-selected depth. Candidates were instructed to jump after researchers verbally gave a 3-2-1 countdown. A trial was unsuccessful and redone if the candidate failed to land within the confines of the force plates. Data collected from SPARTA™ were processed using SPARTA™ Software (v0.12.4), that further calculated metric values (i.e., load, explode, drive). In addition, SPARTA™ outputs composite scores (MSKI Health score, SPARTA™ score, and Risk Group) (Table 1) that are calculated by the normative force plate variables (Table 2).

**TABLE 1:** Definitions of composite scores in DARI™ and SPARTA™.

Composite scores	Definition
SPARTA^TM^ Force Plates	
MSKI Health Score	Version 1 of SPARTA™ Machine learning algorithm trained on SPARTA™ database to predict MSKI
Risk Group	Version 2 of SPARTA™ Machine learning algorithm trained on SPARTA™ database to predict MSKI
SPARTA™ Score	Component score of Load, Explode and Drive
DARI^TM^ Markerless Motion Capture	
Readiness Score	Average of Quality Score Overall and Performance Score Overall
Quality Score	Quality score component *via* joint kinematic and kinetic measurements across all movements
Performance Score	Performance score component *via* center of mass excursion on squat and jump movements

Composite scores represented as, higher = increased performance and decreased MSKI, risk; *MSKI, Risk group* bins subjects 1 to 5, higher score is higher risk, and lower score is lower risk.

**TABLE 2:** Definitions of SPARTA™ and DARI™ component variables.

Component variables	Definition
SPARTA^TM^ Force Plates	
Load (Avg. braking RFD) (N/s)	Average rate of force change from start of braking to start of concentric phase
Explode (Avg. relative concentric force) (N/kg)	Average force between start of concentric phase to liftoff relative to body mass
Drive (Relative concentric impulse) (Ns/kg)	Concentric impulse relative to body mass
Jump Height (m)	Max vertical jump height
Eccentric rate of acceleration (m/s^3^)	Max rate of acceleration in eccentric phase
Max acceleration (m/s^2^)	Peak acceleration
Eccentric impulse (N.s)	Integral of acceleration over eccentric phase
Concentric impulse (N.s)	Integral of acceleration over concentric phase
Max velocity (m/s)	Max velocity of center of mass
Max power (W)	Max of acceleration multiplied by velocity
Unweighting time (s)	Time from unloading start to eccentric start
Eccentric time (s)	Time from start of eccentric to start of concentric phase
Concentric time (s)	Time from start of concentric phase to liftoff
Time to take off (s)	Time from unloaded to liftoff
Time to max acceleration (s)	Time from unload start to max acceleration
Depth (m)	Max depth in the loading phase
Reactive strength index	Jump height divided by time to peak force
Flight time (s)	Time off the force plate
Body Weight (N)	The total mass of the individual during the quiet phase
DARI^TM^ Markerless Motion Capture	
Jump height	Vertical jump height divided by leg length
Squat depth	Squat depth divided by leg length
Hip mobility	Mobility component score *via* squat, overhead squat, and lateral lunge
Hip kinetics	Kinetic component score *via* vertical jump, single leg jump, and multi-hop
Knee mobility	Mobility component score *via* squat, overhead squat, and lateral lunge
Knee kinetics	Kinetic component score *via* vertical jump, single leg jump, and multi-hop
Knee alignment loading	Dynamic valgus loading component score *via* squat, overhead squat, lateral lunge, vertical jump, single leg jump, and multi-hop
Knee alignment landing	Dynamic valgus landing component score *via* vertical jump, single leg jump, and drop jump
Ankle mobility	Mobility component score *via* squat and overhead squat
Spine mobility	Thoracic rotation component score during the reverse lunge with rotation

SPARTA™^,^ Force plate measures normalized to SPARTA™^,^ internal subject database; DARI™^,^ measures normalized to DARI™^,^ internal subject database. Data from each measurement is presented as a normalized score.

DARI™ mMoCap (DARI Motion™, Inc. Overland Park, KS), a 3-dimensional mMoCap system, was used for data collection. Eight Black-fly FLIR GigE cameras (50 Hz) were placed around a 2.5 × 3.5 m matted area. Prior to daily testing, the DARI™ mMoCap was calibrated to the manufacturer’s specifications. DARI™ mMoCap uses Captury Live™ motion tracking software (Captury Live™, The Captury Ltd., Saarbrücken, Germany) that calculates sums of spatial Gaussian functions to generate a subject-specific body model representing the shape and color statistics to estimate joint centers (Stoll et al., 2011). Before capture, a background subtraction was performed on the DARI™ mMoCap system so that when the candidates enters the mMoCap area, the candidates are differentiated from the background during initialization of the tracking model. Candidates were cued into a calibration position, in which both elbows were at 90°, and hands downwards. A computerized subject-based model was generated and virtually overlaid on the live image of the candidates, and scaling actions (lunges, squats, arm rotations) were performed to capture the candidates joint centers (Cabarkapa et al., 2022). Candidates performed the DARI™ movement screen which consisted of reverse lunge with rotation, lateral lunge, body weight squat, overhead squat, CMJ, drop jump, single leg CMJ, and five consecutive single leg hops. All unilateral movements were performed twice (right and left limb), and bilateral movements were performed once, except for body weight squat and CMJ which were performed three times. The drop jump height was standardized at 18 inches for each candidate. The movement screen was built with the manufacture’s recommendations to evaluate for lower extremity and torso movements. All movements were demonstrated, cued by the researcher, and were performed on a 3-2-1 countdown before the initiation of the candidate’s movement with ∼15 s between movements. If the skeleton was visually misaligned from a joint center, either the candidates would redo the movement, or the skeleton would be re-tracked *post hoc*. Skelton’s were re-tracked automatically *post hoc* by the manufacturer’s recommendations *via* the proprietary software, Captury Live™ motion tracking software, described previously. All variables from DARI™ mMoCap were uploaded to DARI’s™ cloud platform and processed using DARI™ Insight Processing (version 1.0.4-250) and DARI™ Insight Vault (version 1.0.3-854) software. mMoCap joint coordinate systems are defined during movement tracking and calculations for knee, hip and ankle kinematics following the methods prescribed by the International Society of Biomechanics (Grood and Suntay, 1983; Wu et al., 2005). DARI™ mMoCap automatically calculates composite scores (Readiness Score, Quality score, and Performance Score) (Table 1), and normalized component variables (e.g., Hip mobility, Knee kinetics) (Table 2) when a screening test is complete.

It is important to note that composite scores are calculated *via* the component scores for both DARI™ and SPARTA™. The composite scores are proprietary calculations, thus considered “black box” algorithms due to the lack of transparency in which these are derived. DARI’s™ component scores are aggregates of kinematic and kinetic variables in different movements through the DARI™ screen. While SPARTA’s™ component scores are aggregates of the raw force time curve that calculate into kinetic and kinematic variables. All measurements (composite and component scores) are considered arbitrary as they are further normalized or aggregates of the normalized scores. Both DARI™ and SPARTA™ composite and component scores are represented where, higher scores are better performance or lesser MSKI risk. While SPARTA™ Risk Group score is a binned measure, where five denotes greater MSKI risk and one is less MSKI risk.

### Data analysis

Independent sample t-tests were used to compare the differences of age, weight, and height separately in male and female candidates. De-identified MSKIs were collected from the OCS internal reports for candidates that presented to the OCS Physical Therapy department for treatment during the 10 weeks of training. MSKIs were labeled by anatomic location: 1) lower body (foot, ankle, knee, lower leg, and upper leg) 2) torso (lumbar spine, thoracic spine, ribs, and hip), 3) upper body (shoulder, elbow, upper arm, forearm, hand, and wrist), 4) head and neck (cervical spine). The outcome variable was labeled as MSKI or noMSKI. Inclusion criteria for a MSKI was lower body or torso, and noMSKI were labeled as not receiving a MSKI or upper body and head and neck. Self-reported prior injury had the same classification as the outcome variable (MSKI or noMSKI).

*Analysis one* included estimation of two binary logistic regression model. An unadjusted model was first used to determine if any of the explanatory variables (common MSKI risk factors (cMSKI), SPARTA™, and DARI™ composite scores) predicted lower extremity and torso MSKI in candidates (Table 1). The adjusted model controlled for the effect of cMSKI variables (sex, age, and three mile run time) when testing whether the DARI™ and SPARTA™ composite scores predicted MSKI. Statistical analyses were conducted using IBM SPSS Statistics Version 25 (IBM Corp; Armonk, NY). Statistical significance was set *a priori* at *α* = .05, two-sided.

**FIGURE 1:**
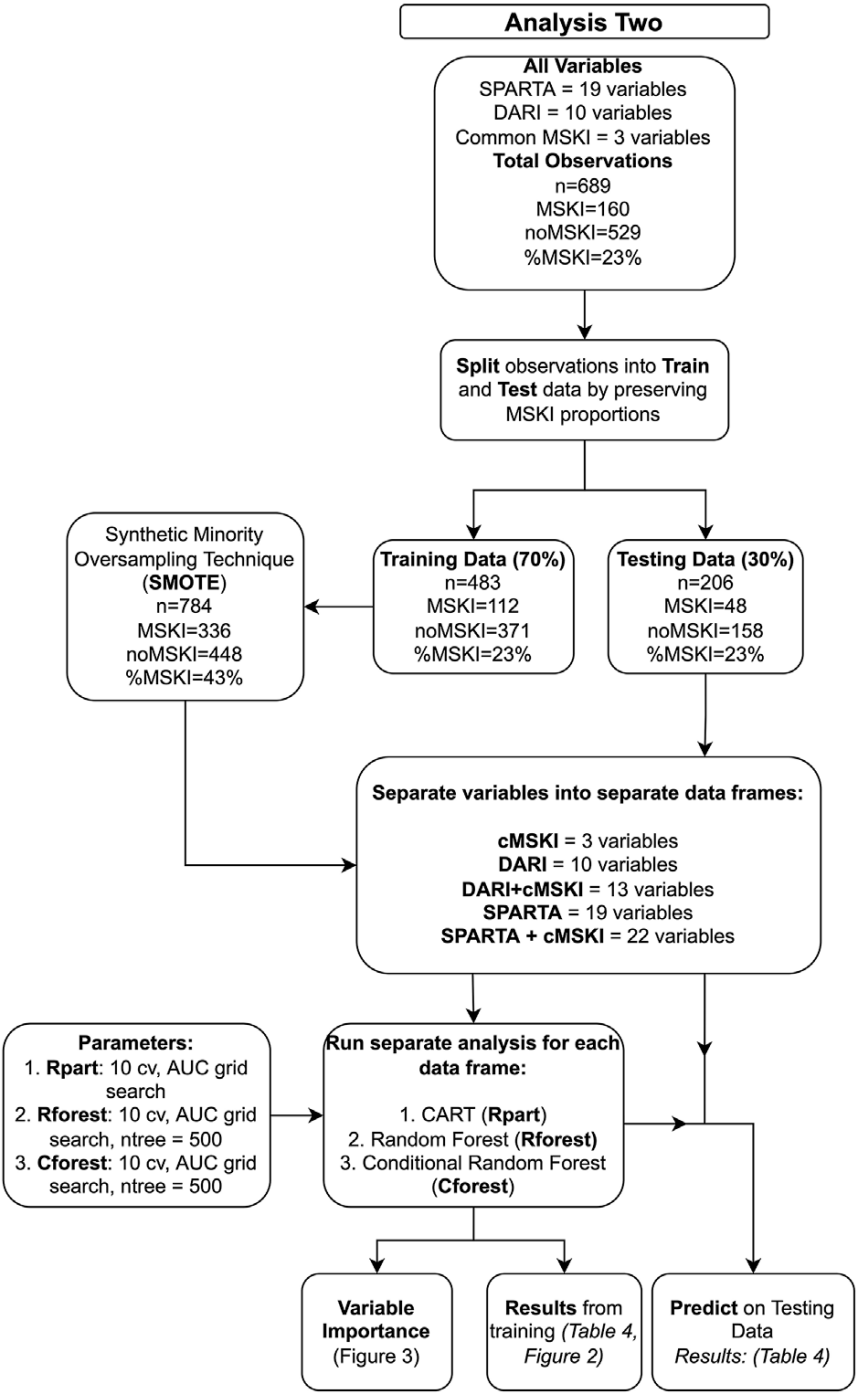
Data analysis pipeline for *analysis two* on the normalized component variables.

*Analysis two* (Figure 1) evaluated SPARTA™ and DARI™ component variables (Table 2) that were used to calculate SPARTA™ and DARI™ composite scores in analysis one (Table 1). The data was split into a train (70%) and test (30%) with the same proportions of MSKIs in each train and test set for both the SPARTA™ and DARI™ data sets using “createDataPartition” function (Caret, v. 6.0). Due to imbalanced MSKIs, Synthetic Minority Oversampling Technique (SMOTE, DMwR, v 0.4.1), was used on the training data set. Since MSKIs are infrequent when compared to noMSKI, oversampling, down sampling or synthesizing new minority variables may be a technique to increase the prevalence of observations with a MSKI (Carey et al., 2017; Fernández et al., 2018). Recursive partitioning and regression trees (Rpart), random forest (Rforest) and conditional random forest (Cforest) were used to model the training data with a ten-fold cross-validation and grid search for the greatest AUC value (caret, v 6.0-93). In Rforest and Cforest, the grid search evaluates different variables tried at each split (mtry) and in Rpart the grid search evaluates the complexity parameter (cp) in which the highest AUC in accordance with mtry and/or cp was chosen as the final model. A total of three algorithms were used (1. Rpart, 2. Rforest, 3. Cforest) to train on five data sets (1. cMSKI, 2. DARI™ + cMSKI, 3. DARI™, 4. SPARTA™ + cMSKI, and 5. SPARTA™), totaling to 15 final training models. The 15 final models were tested on their respective test set (30%) using the “predict” function along with “confusionMatrix” function (caret v. 6.0) to assess the accuracy, specificity, sensitivity/recall, precision, and F1 score. All analyses in aim two were conducted using R Version 3.6.1 (R Core Team, 2019). Since classes were near balanced utilizing SMOTE, the probability threshold was set at ≥0.50 for MSKI and <0.50 for noMSKI for classification of model performance.

Measures relating to precision, F1 score, sensitivity and other performance measures will be described to demonstrate the model performance at the previously stated probability threshold (≥.50 = MSKI and <.50 = noMSKI). Although, as described in Ruddy et al. (2018), AUC may be a better performance outcome to report, since AUC is an aggregate of the true positive and false positive rates across all classification thresholds among the receiver operating characteristic (ROC) curve. In clinical practice, AUC is described as the probability a randomly chosen MSKI candidate is ranked more likely to have a MSKI than a randomly chosen noMSKI candidate (Hanley and Mcneil, 1982; Hajian-Tilaki, 2013). AUC performance were classified as .50–.60 are by chance, .61–0.70 poor, .71–.80 fair, .81–.90 good, and .91–1.00 excellent (Fawcett, 2004; Karuc et al., 2021). Thus, if a model scored an AUC of 0.50 this model would have the same probability as to flipping a coin (50/50), “by chance”.

### Decision tree models and variable importance

Rpart, Rforest and Cforest are all recursive partitioning methods where decision trees are constructed to classify observations *via* independent variables (Loh, 2011). Rpart is a single decision tree algorithm that utilizes binary splits, where the splitting criteria at each split is determined by Gini impurity, in which each split attempts to maximize purity. Rforest uses many decision trees and for each tree a random number of variables will be tried at each split and takes the majority vote across all trees for prediction (Breiman, 2001). Similarly, Rforest utilizes the same splitting criterion as Rpart, Gini impurity. Cforest functions similarly to Rforest although, Cforest default parameters utilize subsampling without replacement found in the “Party” package, instead of bootstrapping with replacement found in Rforest default parameters (Strobl et al., 2009). In addition, Cforest utilizes conditional inference trees as base learners. The conditional inference trees use the significance tests for variable selection and to find the optimal binary splits, rather than Gini impurity (Strobl et al., 2007). Cforest implementation of unbiased approaches may allow for increased interpretation of variable importance by treating categorical and continuous unbiased. Variable importance for each model was calculated by the base packages using the caret package with a scaling factor, based on the recommendations provided by Strobl et al. (2009), *Rpart*: Gini Importance, *Rforest*: Gini Importance and Permutation Importance, *Cforest*: Permutation Importance. Gini importance across Rpart and Rforest utilize the basic properties of Gini index splitting criterion. While permutation importance found in Cforest and Rforest randomly permutates predictor variables with the out-of-bag observations and assess the decrease in accuracy. Further information regarding variable importance in Rpart, Rforest and Cforest can be found in the R documentation (R Core Team, 2019; Strobl et al., 2007; Strobl, 2008; Strobl et al., 2009).

## Results

Female candidates had less mass than male candidates (Female candidates = 64.7 ± 6.7 kg; Male candidates = 80.1 ± 9.2 kg, Cohn’s *d =* 1.740), and were significantly shorter (Female candidates = 164.0 ± 5.6 cm; Male candidates; 176.5 ± 6.8 cm, Cohn’s *d =* 1.853), while there was no difference in age (Female candidates = 24.7 ± 3.1 years; Male candidates = 24.9 ± 3.0 years, Cohn’s *d =* 0.072).

Simple logistic regression analyses demonstrated that, when analyzed separately, the cMSKI variables: sex (*p* = 0.002), age (*p* < 0.001) and three-mile run (*p* < 0.001) time were significant predictors of MSKI (Table 3). Odds Ratios demonstrated that female candidates were 2.0x more likely to suffer an MSKI than male candidates, each one unit increase in age increased the likelihood of MSKI by 14%, and every added minute of run time increased MSKI likelihood by 19% (Table 3). For SPARTA™ outputs, MSKI Health Score was a significant predictor of MSKI such that every one unit increase in MSKI Health Score decreased the likelihood of MSKI by 4.7%, while Risk group (Omnibus *p*-value = .347) and SPARTA™ Score were not significant predictors of MSKI (Table 3). For DARI™, Readiness and Performance scores were significant predictors of MSKI, but Quality score was not. For every one unit increase in Readiness and Performance score, the likelihood of MSKI decreased by 1.8% and 1.2%, respectively (Table 3). Despite statistical significance, when AUC was calculated on SPARTA™ and DARI™ composite scores, MSKI Health score (AUC = .57), Readiness score (AUC = .55) and Performance score (AUC = .56) were poor classifiers of MSKI and noMSKI. Additionally, when SPARTA™ and DARI™ were adjusted for the significant cMSKI variables (sex, age, 3-mile run times), no SPARTA™ or DARI™ composite scores were significant predictors of MSKI (Table 3), except for the SPARTA™ MSKI Health score.

**TABLE 3:** Simple and multiple logistic regression.

Predictor	Group	MSKI (Mean ± SD) n = 160	noMSKI (Mean ± SD) n = 529	OR (95% CI)	*p*-value (simple logistic regression)	Adjusted OR (95% CI)	*p*-value (multiple logistic regression)
Common MSKI Risk Factors (cMSKI)							
Sex (% Female)	—	42/160 = 26.3%	81/529 = 15.3%	**1.969(1.288, 3.009)**	**.002**	—	—
BMI	—	25.12 ± 2.32	25.47 ± 2.23	0.932 (0.861, 1.008)	.079	—	—
Age (years)	—	25.84 ± 3.44	24.58 ± 2.84	**1.140(1.077, 1.206)**	**<.001**	—	—
Prior injury history (% Prior injury)	—	11/160 = 6.9%	20/529 = 3.8%	1.879 (.880, 3.822)	.103	—	—
3-mile run time (min)	—	21.81 ± 2.10	21.03 ± 2.03	**1.192(1.096, 1.296)**	**<.001**	—	—
SPARTA^TM^ Force Plates							
MSKI Health Score	—	55.52 ± 5.31	56.73 ± 4.94	**.953 (.919, .988)**	**.009**	**.959 (.924, .995)**	**.025**
Risk Group	1 (Low-risk) (Reference)	65/160 = 40.6%	192/529 = 36.3%	—	—	—	—
	2	33/160 = 20.6%	119/529 = 22.5%	.819 (.508,1.320)	.413	.946 (.559, 1.600)	.83**6**
	3	31/160 = 19.4%	97/529 = 18.3%	.944 (.577,1.545)	.819	1.048 (.613, 1.790)	.864
	4	25/160 = 15.6%	79/529 = 14.9%	.935 (.550,1.589)	.803	1.170 (.658, 2.080)	.592
	5(High-risk)	6/160 = 3.8%	42/529 = 7.9%	.422 (.171,1.038)	.060	.457 (.180, 1.162)	.100
SPARTA™ Score	—	78.10 ± 3.59	78.50 ± 3.86	.972 (.927, 1.019)	.238	.968 (.919, 1.019)	.210
DARI^TM^ Markerless Motion Capture							
Readiness Score	—	55.10 ± 12.37	57.45 ± 11.00	**.982 (.967, .997)**	**.020**	.993 (0.976, 1.011)	.464
Quality Score	—	42.42 ± 4.72	42.36 ± 4.77	1.003 (.966, 1.041)	.880	1.016 (.977, 1.057)	.421
Performance Score	—	67.70 ± 22.00	72.55 ± 19.09	**.988(.980, .997)**	**.007**	.994 (.984, 1.005)	.289

Common MSKI, risk factors, SPARTA™ FPs, and DARI™ mMoCap composite scores; Data are presented as descriptive statistics and results of the simple logistic regression (unadjusted) and multiple logistic regression (adjusted for sex, age, and three mile run time).

Bold values denote significant.

**FIGURE 2:**
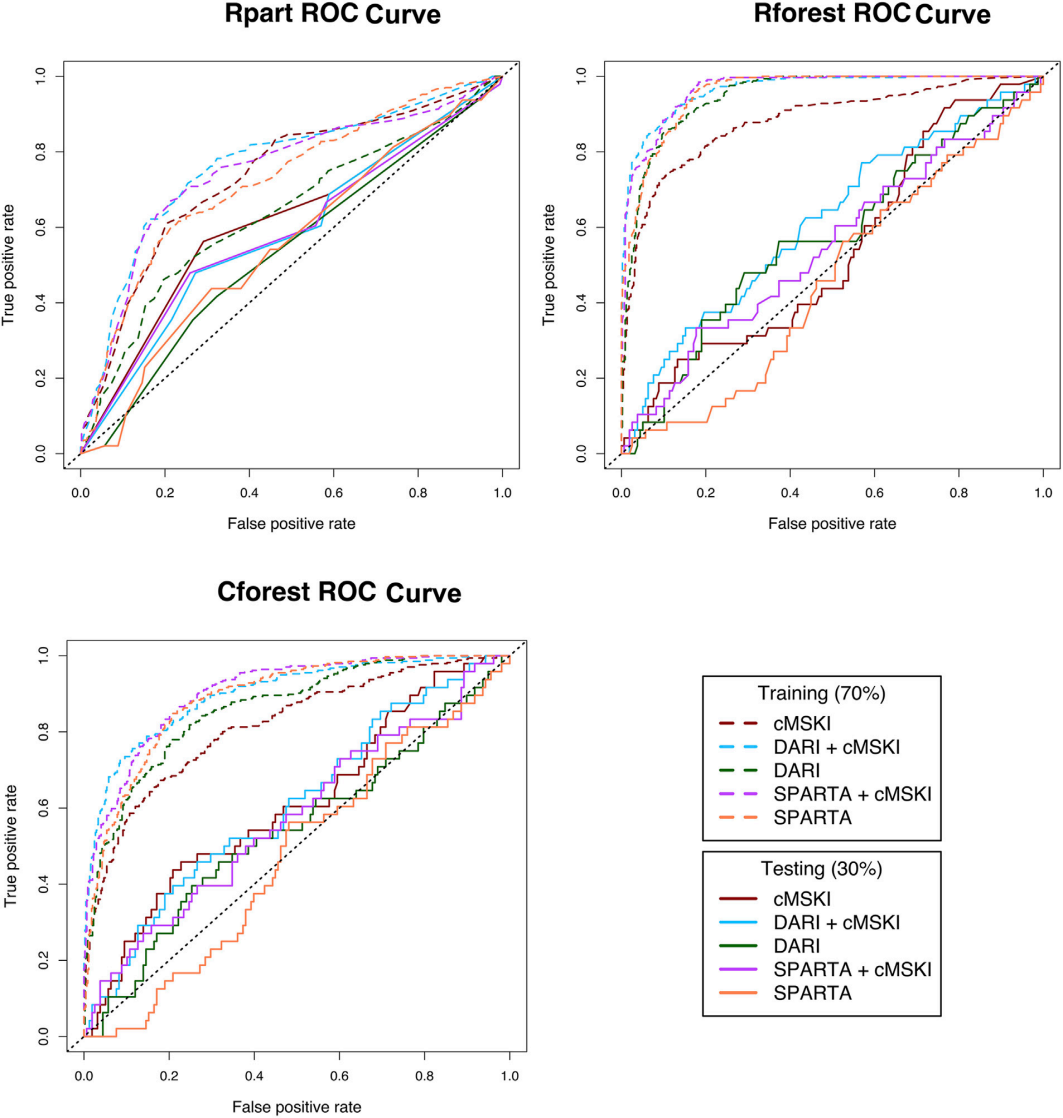
Receiver operating characteristic (ROC) curves for Rpart, Rforest, and Cforest algorthiums training and testing data; Rpart, Cforest, and Rforest algorithums were trained and tested on the different data sets: 1) Common muskuloskeltal injury (cMSKI), 2) DARI™ + cMSKI, 3) DARI™, 4) SPARTA™ + cMSKI, and 5)SPARTA™; Area under the curve (AUC) is calculated for each ROC curve in Table 4.

*Analysis two* (Figure 1) evaluated whether the component scores (Table 2) underlying calculations of SPARTA™ and DARI™ composite scores (Table 1) were associated with MSKI risk during OCS. SPARTA™, DARI™ and cMSKI (significant predictors from analysis one: sex, age, three-mile run time) variables were merged. The entire data frame was split into training (70%, *n* = 483, MSKI = 112, noMSKI = 371, %MSKI = 23%) and test (30%, *n* = 206, MSKI = 48, noMSKI = 158, and %MSKI = 23%) data sets. SMOTE was performed on the training set and increased the total number of observations (*n* = 784, MSKI = 336, noMSKI = 448, %MSKI = 43%). Rpart, Rforest, and Cforest were run separately on the cMSKI (3 variables), DARI™ + cMSKI (13 variables), DARI™ (10 variables), SPARTA™ + cMSKI (22 variables), and SPARTA™ (19 variables) with the same set of observations. Results from the training and testing data for each data frame and algorithm (Rpart, Rforest, and Cforest) are listed in Table 4. Overall training AUC performance for Rpart ranged from .64 to .75, Rforest .88 to .97, and Cforest .82 to .90. While testing AUC performance for Rpart, ranged from .54 to .61, Rforest .46 to .62, and Cforest .47 to .61 (Table 4; Figure 2).

**TABLE 4:** Training and Testing Model Performance Across all Algorithms and Data sets.

		Training (*n* = 784)						Testing (*n* = 205)					
		cMSKI	Dari™	Dari™ + cMSKI	SPARTA™	SPART™ + cMSKI	Average	cMSKI	Dari™	Dari™ + cMSKI	SPARTA™	SPARTA™ + cMSKI	Average
Rpart	Accuracy	.72	.65	.74	.70	.74	**.71**	.67	.65	.68	.58	.68	**.65**
	Sensitivity/Recall	.61	.47	.62	.61	.67	**.60**	.56	.35	.35	.44	.48	**.44**
	Specificity	.80	.79	.84	.77	.79	**.80**	.71	.73	.78	.62	.74	**.72**
	AUC	**.74**	**.64**	**.77**	**.72**	**.75**	**.72**	**.61**	**.54**	**.58**	**.55**	**.58**	**.57**
	Precision	.69	.63	.74	.67	.71	**.69**	.37	.29	.33	.26	.36	**.32**
	F1 Score	.65	.54	.68	.64	.69	**.64**	.45	.32	.34	.33	.41	**.37**
Rforest	Accuracy	.82	.87	.89	.87	.89	**.87**	.58	.67	.70	.54	.61	**.62**
	Sensitivity/Recall	.68	.81	.82	.86	.86	**.80**	.33	.38	.38	.33	.35	**.35**
	Specificity	.92	.92	.95	.87	.90	**.91**	.66	.75	.80	.60	.68	**.70**
	AUC	**.88**	**.95**	**.97**	**.96**	**.97**	**.94**	**.54**	**.57**	**.62**	**.46**	**.55**	**.55**
	Precision	.87	.88	.92	.83	.87	**.88**	.23	.32	.36	.20	.25	**.27**
	F1 Score	.76	.84	.87	.85	.87	**.84**	.27	.34	.37	.25	.30	**.31**
Cforest	Accuracy	.76	.78	.83	.80	.81	**.79**	.67	.67	.70	.54	.64	**.64**
	Sensitivity/Recall	.63	.63	.69	.69	.70	**.67**	.46	.35	.31	.38	.40	**.38**
	Specificity	.85	.90	.92	.87	.89	**.89**	.73	.76	.82	.59	.71	**.72**
	AUC	**.82**	**.86**	**.90**	**.89**	**.90**	**.87**	**.61**	**.54**	**.61**	**.47**	**.57**	**.56**
	Precision	.76	.82	.87	.80	.83	**.82**	.34	.31	.35	.22	.29	**.30**
	F1 Score	.69	.71	.77	.74	.76	**.74**	.39	.33	.33	.28	.34	**.33**
Average	Accuracy	.76	.77	.82	.79	.81	**.79**	.64	.66	.70	.55	.64	**.64**
	Sensitivity/Recall	.64	.63	.71	.72	.75	**.69**	.45	.36	.35	.38	.41	**.39**
	Specificity	.86	.87	.90	.84	.86	**.87**	.70	.75	.80	.61	.71	**.71**
	AUC	**.81**	**.82**	**.88**	**.86**	**.87**	**.85**	**.59**	**.55**	**.60**	**.50**	**.57**	**.56**
	Precision	.78	.78	.85	.77	.80	**.79**	.31	.30	.35	.23	.30	**.30**
	F1 Score	.70	.70	.77	.74	.77	**.74**	.37	.33	.35	.28	.35	**.34**

Describes *Analysis two.* Training model performance for *n* = 784 after SMOTE., Separate data frames (cMSKI, DARI™, DARI™ + cMSKI, SPARTA™, SPARTA™ + cMSKI, 5 data frames) modeled by different algorithms (Recursive partitioning and regression trees (Rpart), random forest (Rforest), and conditional random forest (Cforest) = 3 algorithms), totaling to 15 separate analysis. Each analysis tested on testing data (*n* = 205). Area under curve (AUC) = total model performance. All other measures using threshold probability of ≥0.50 = MSKI, and <0.50 = noMSKI, for model performance classification.

Bold values denote AUC values.

When cMSKI variables were trained alone, the training models performed fair to good (AUC = Rpart: .74, Rforest: .88, Cforest: .82) and performed slightly better than DARI™ and SPARTA™ alone in Rpart, while lesser in Rforest and Cforest training models. When cMSKI variables were tested, AUC performance was similar to all other training models and performed by chance or poor (AUC = Rpart: .61, Rforest: .57, Cforest: 0.61). In addition, when cMSKI variables were added to DARI™ and SPARTA™, AUC model performance increased slightly in both the training and testing. When comparing the training algorithms averaged across the different data frames, Rforest performed the best (AUC avg = .94), than Cforest (AUC avg = .87), and then Rpart (AUC avg = .72). Interestingly when tested, AUC averaged across the data frames was similar between the algorithms Rpart (.57), Rforest (.55), and Cforest (.56) (Table 4; Figure 2). Measures of specific model performance (accuracy, specificity, sensitivity) are presented in Table 4 with the threshold of ≥.50 for MSKI and <.50 for noMSKI.

**FIGURE 3:**
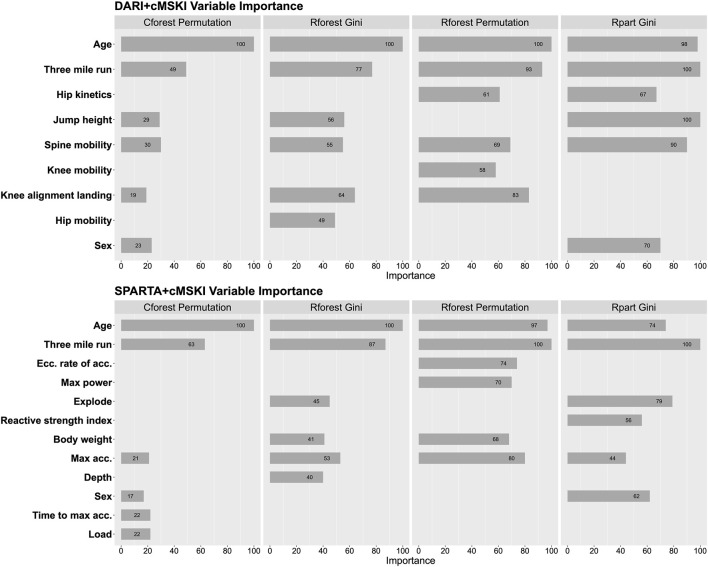
Feature importance for Rpart Gini, Rforest Gini and Permutation, and Cforest Permutation; Calculated on DARI™ + cMSKI and SPARTA™ + cMSKI.

Global variable importance was analyzed for each algorithm for DARI™ + cMSKI and SPARTA™ + cMSKI. Results demonstrate that age, three mile run time, and spine mobility had a level of importance across all algorithms in DARI™ + cMSKI, while in SPARTA™ + cMSKI age, three mile run time, and max acceleration had a level of importance in all algorithms. Lastly, sex had a level of importance in only Cforest permutation and Rpart Gini for both DARI™ + cMSKI and SPARTA™ + cMSKI (Figure 3).

## Discussion

This study evaluated the utility of commercial grade technology composite scores to predict MSKI during Marine Corps Officer Candidates School. Both SPARTA™ (i.e., MSKI health score) and DARI™ (i.e., Readiness, Performance scores) composite scores were predictive of lower extremity and torso MSKI, although their clinical utility may be limited (Table 3). When component variables (Table 2) were trained using different supervised machine learning algorithms with a 10-fold cross validation, AUC performance averaged across the data frame for Rforest, Cforest, and Rpart were excellent (.94), good (.87), and fair (.72), respectively. When the trained models were subsequently tested on the testing data, performance was by chance and similar averaged across algorithms (AUC avg = Rpart: .57, Rforest: .55, Cforest: .56). In addition, a model consisting of only cMSKI variables (age, sex, and 3-mile run time) when tested, performed better than DARI™ and SPARTA™ alone in Rpart and Cforest algorithms. The addition of cMSKI variables to SPARTA™ and DARI™ (DARI™ + cMSKI and SPARTA™ + cMSKI) testing models slightly increased AUC performance but remained poor, overall. This is the first study to evaluate the SPARTA™ and DARI™ component variables (Table 2) in addition to the proprietary composite scores and further demonstrated that DARI™ and SPARTA™ do not provide greater predictive ability for MSKI than commonly assessed cMSKI variables, such as age, sex, and 3-mile run time.

When cMSKI variables were analyzed (Table 3), results were as expected in which slower run times, female sex, and older age were associated with greater risk for developing a MSKI. These results are comparable to Army Basic Combat Training, as females had twice the injury rates of males (Knapik et al., 2001), older military members were more likely to get injured [Age >25, OR (CI) = 1.83 (1.75,1.91)] (Sulsky et al., 2018), and slower run times [Males >19.21 min, OR (CI) = 1.6 (1.0–2.4), and females >23.49 min, OR (CI) = 1.9 (1.2–2.8)] were associated with greater MSKI risk (Knapik et al., 2001). In addition, Hando et al. (2022) reported that in Air Force special warfare trainees (USAF SW trainees), cMSKI variables (i.e., slower run time and older age) were associated with greater MSKI risk, as well as previous MSKI. In our findings, self-reported previous MSKI was not a significant predictor in candidates, but this may be due to the self-report nature and candidates non-willingness to disclose or report a prior injury. The non-willingness to disclose an injury may be due to the candidates not reporting the injury to military leadership prior to OCS, in which may be a potential of dismissal from the course (e.g., surgery). Although the data was de-identified and poses minimal risk to the candidates, self-reported questionnaires regarding MSKIs during OCS may be an un-reliable source of data for future studies.

With the integration of the high-performance model for sport into military settings, it is necessary that research efforts test the efficacy of commercial technologies within the specific population. Currently, there is limited evidence to support the predictive ability of SPARTA™ or DARI™ composite scores for MSKI in military trainees. This is further described in Hando et al. (2021); Hando et al. (2022) wherein SPARTA™ MSKI Health score had no predictive utility and DARI™ Quality score had limited utility to determine MSKI risk in USAF SW trainees. However, military training environments are diverse and can vary by sex, MSKI proportions, MSKI surveillance period, training type and military branch. For example, Hando et al. reported 37.4% and 28% of male trainees incurred “any MSKI” and “lower extremity MSKI” respectively in an 8-week period. When compared to Marine Officer candidates, 23% of male and female candidates incurred a torso or lower extremity MSKI in a 10-week period. Such differences further demonstrate that each use case within military training environments should be independently investigated towards the specific population of interest.

As shown, each population is unique and the training data SPARTA™ uses for a predictive MSKI Health score may not be directly applicable to USAF SW trainees, whereas candidates may have been similar to the SPARTA™ training data set. Although, when evaluating AUC performance for the SPARTA™ MSKI Health score, results were similar to Hando et al. (2022) (candidates: AUC = .57; USAF SW: AUC = .52 and .51), thus SPARTA™ MSKI Health score were not clinically relevant and had limited utility in identifying candidates at risk for MSKI, even though a statistically significant predictor (*p* = .009). DARI™ composite scores (i.e., Readi ness score) also had similar outcomes as SPARTA™ MSKI health score (DARI™: *p* = .020; AUC = .55) in which they were statistically significant but not clinically relevant for MSKI. Interestingly, the SPARTA™ Risk Group measure had an unexpected inverse trend in which risk group 5 (High risk), was proportionally the least likely to develop a MSKI as demonstrated in Table 3. This data demonstrates that composite scores were predictive, except SPARTA™ Risk Group, but classification of MSKI risk was no better than “by chance”. Furthermore, when deciding what commercial grade MSKI machine learning model and screening measures to implement it is important to understand 1) what population normative ranges the model was trained on and 2) if the screening measures are applicable to the population of interest. With these considerations a within population specific analysis may be deemed more appropriate to build machine learning models for deployment. Although not a supervised approach, candidates were stratified within the population using unsupervised learning on the CMJ and had a strong association to MSKI risk in low and high performers with overlap of MSKI and noMSKI within these groups (Bird et al., 2022). Lastly, this demonstrates that CMJs on force plates may be a useful screening tool, but MSKI risk is not fully explained by a CMJ on a force plate.

The *second aim* of the analysis was to train a model on the candidates with the normalized component variables (Table 2) that calculate the DARI™ and SPARTA™ composite scores (Table 1), and then evaluate if the addition of cMSKI variables (sex, age, and 3-mile run time) increased performance of SPARTA™ and DARI™ using different supervised machine learning algorithms (Rpart, Cforest, and Rforest). Both DARI™ and SPARTA™ component variables performed similarly on the training and testing data averaged across algorithms (Training AUC avg.: DARI™ = .82, SPARTA™ = 0.86; Test AUC avg: DARI™ = .55, SPARTA™ = .50) (Table 4). Described in Table 4, when cMSKI variables were added to DARI™ and SPARTA™, both training and testing AUC avg. Increased slightly (∆: .01–.07). Overall, cMSKI variables added predictive utility to DARI™ and SPARTA™ but testing demonstrated “by chance” and “poor” predictive utility. These results compare to other predictive MSKI risk screening studies, where Nordic hamstring strength and demographics could not be used to predict hamstring strains in elite footballers (Random forest median AUC = .52 and .53) (Ruddy et al., 2018). In addition, functional movement tests, force plate testing, and demographics demonstrated poor injury prediction (Decision tree ensembles AUC = .663, sensitivity = 55.6%) in elite male youth football players (Oliver et al., 2020). Lastly, Functional Movement Screen (FMS) and demographics demonstrated prediction by chance in a non-athletic group (Naïve Bayes AUC: .58) and poor prediction in the athletic group (logistic regression AUC: .63) (Karuc et al., 2021). Although these studies included a “risk factor” task (i.e., FMS) with demographics, multiple physiological domains were not tested. Rommers et al. (2020) successfully predicted injury using a multi-battery physiological and sport domain testing (i.e., aerobic, anaerobic, power, and sport specific skill tests) and basic demographics (F1-score = 85%, sensitivity = 85%) in elite youth football players, but did not report AUC. In addition, Thornton et al. (2017) demonstrated that training load (i.e., rating of perceived exertion, total distance, high-speed running distance) could appropriately predict injury (Random forest AUC: .74). Discussed in the review by Bittencourt et al. (2016) injuries are multi-faceted and arise from a web of determinants and not from isolated predictive factors. Such data supports the use of a multi-domain testing battery rather than individual tests for injury prediction. In addition, screening measures alone may not demonstrate the causal relationship to the onset of injury, as the tasks performed leading to the injury (demonstrated by training load) may be a necessary additive predictor to an injury risk model. While modeling diverse predictors, it is also important to evaluate the type of algorithms used for modeling. We used different recursive and partitioning methods from simple (Rpart) to more complex black box algorithms (Cforest and Rforest).

When training Rforest models, AUC performance was excellent for all data frames except cMSKI variables alone (AUC = .88). While Cforest and Rpart AUC averaged across data frames performance were good and fair, respectively. Then when the training models were tested on the respective testing set, AUC performance averaged across data frames were classified by chance between Rpart, Cforest, and Rforest. In addition, when cMSKI training models were trained the AUC performance averaged across Rpart, Rforest, and Cforest (AUC avg = 0.81) was less than the inclusion of DARI™ and SPARTA™ data frames. Interestingly, the AUC performance averaged across Rpart, Rforest and Cforest (AUC avg = 0.59), was the second highest AUC between the data frames. These results demonstrate the potential overfitting in Rforest and Cforest compared to Rpart when the testing AUC performance demonstrated similar results. Even with the large cohort of subjects in this current study, modeling approaches using complex decision trees (i.e., random forest) presented major limitations in providing over optimization in the training performance results.

These limitations provide a theoretical framework proposed by William of Occam, Occam’s razor where a simple solution is preferred (Blumer et al., 1987). Thus, an interpretable single decision tree model (i.e., Rpart) may be of greater value to add to an organization to evaluate “under the hood” model performance rather than a black box model (i.e., random forest). In addition, since demographics are less prone to change (i.e., age increases 1 unit yearly), utilizing this technology serially (weekly, monthly) may add a benefit in increasing the likelihood of predicting MSKI risk with simple modeling approaches, as alluded to previously regarding training load. Lastly, this is theorized as a dynamic systems approach where athletes are like hurricanes, a non-linear dynamic system (Stern et al., 2020). These findings don’t dismiss these technologies to predict MSKI, but they do question the utility in screening once over a long exposure (10 weeks of training). Future investigations should evaluate serial screening tests that may increase the sensitivity of MSKI prediction, since SPARTA™ FP and DARI™ mMoCap demonstrated excellent reliability (SPARTA™ ICC >.90; DARI™ ICC >.80) (Cabarkapa et al., 2022; Hando et al., 2022).

Regarding variable importance (Figure 3), we demonstrated age and 3-mile run time were the top selected variables across all algorithms for DARI™ + cMSKI and SPARTA™ + cMSKI. Interestingly, sex was only chosen for Cforest permutation and Rpart gini, although it is a significant factor for increased likelihood of MSKI for females candidates demonstrated in Table 3. This may be due to the limitations of variable importance in Rforest approaches (Strobl et al., 2007; Strobl et al., 2009). In approaches such as Rpart and Rforest, these models may tend to bias continuous variables, rather than a binary categorical variable with only two possible splits in a decision tree. Unbiased approaches such as conditional random forest may decrease this limitation demonstrated in Figure 3, in which sex had some level of importance in Cforest permutation. Thus, we recommend that Rforest be held with caution for variable importance interpretation when utilizing categorical and continuous variables. Lastly, when evaluating the Cforest Permutation variable importance we have demonstrated that cMSKI are the primary variables for describing the models, but the secondary risk factors such as DARI™ and SPARTA™ variables (i.e., spine mobility and max acceleration) should not be ignored as they add value to the model’s increased performance.

We demonstrate model validation in a new testing data set for the composite and component scores. When analyzing the composite scores (i.e., MSKI Health Score), these proprietary models were trained and normalized to SPARTA™ or DARI’s™ internal databases, thus proper validation is necessary in a new population (i.e., candidates). We demonstrated that SPARTA™ and DARI’s™ composite scores were predictive in candidates but provided poor utility in clinical use case. In addition, when a model was trained on the normalized component scores, we allocated a separate test set (30% of data) to validate the trained model’s efficacy in an unseen data set. In the domain of human research, specifically MSKIs with machine learning outcomes, the number of positive outcomes (MSKIs) in addition to the number of subjects needed to test in a multi-battery test is a large limiter to overfitting and bias. While in other fields, there is the luxury to large open-source data sets and millions of observations (Chatzis et al., 2018). Recently, Karnuta et al. (Karnuta et al., 2020) published an epidemiological machine learning analysis on a large cohort of position Major League Baseball players (*n* = 1,931 unique position players and *n* = 1245 unique pitcher players) to predict injuries in an open-source online data base. On the other hand, military injury data and key performance indicators, such as physiological measures, are strategically safe-guarded and not readily accessible. In general, few research teams and practitioners have access to these types of data with a limited cohort of a sub-sample of a military population, thus we recommend the collaborations across institutions necessary to collect large cohorts of varying types of military populations. This is turn would allow for the validation of models between populations for practical prescription use case (Bullock et al., 2022).

To summarize, the large discrepancies between the training and testing AUC performance, and the overall poor testing AUC performance could be a factor of many reasons, 1) overfitting of the training models (specifically in random forest), 2) relatively small sample when compared to other fields (e.g., finance), 3) noise in the outcome variable (e.g., noMSKI did not seek medical attention), and 4) the variables used for modeling does not describe MSKI in candidates. Strengths of this study include the MSKI reporting by the same medical staff through OCS. OCS requires all candidates to perform the same tasks (i.e., hikes, physical fitness, and graded events), thus the training load requirements are similar across all candidates mitigating confounders during the 10 weeks of training. Limitations include the relatively small sample size for female candidates, although this sample size is representative of female candidates that enter through OCS. In addition, since a MSKI classification may be subject for removal of OCS, noMSKI candidates may have not sought out medical attention. Future directions include testing SPARTA™ and DARI™ in varying populations (e.g., athletics, general population) for further validation. Lastly, serial monitoring (testing multiple times through the 10 weeks) and/or continuous monitoring (e.g., heart rate, accelerometry) may be necessary to refine MSKI models.

## Conclusion

In determining the commercial grade system to use in a dynamic military environment we encourage the practitioner to investigate whether the technology has been tested for its utility on the desired outcome measure (i.e., MSKI) in the population of interest. We have demonstrated SPARTA™ (MSKI Health score) and DARI™ (Readiness score and Performance score) are predictive of MSKI, but with limited clinical relevance due to the poor AUC performance. In addition, we demonstrated the normalized component variables in both SPARTA™ and DARI™ have similar predictive utility when trained and tested on the population (Table 4; Figure 2) compared to SPARTA™ and DARI’s™ composite scores (Table 3) and cMSKI variables, while classification *via* AUC performance was “by chance.” While a “one stop shop number” (i.e., Risk Group or Readiness Score) is the striving goal in MSKI risk for actionable decision aids, we have demonstrated single composite scores and a trained model of the normalized component scores have limited utility to predict MSKI over a 10-week of Officer Candidates School in Marines.

This work was funded by The Office of Naval Research (N00014-20-C-2020). Contents are solely the responsibility of the authors and do not necessarily represent the official views of the Department of Army/Navy/Air Force, Department of Defense, or the United States Government.

## Data Availability

The datasets presented in this article are not readily available because of the contracting through the Office of Naval Research. Requests to access the datasets should be directed to Bradley Nindl, bnindl@pitt.edu.
